# Coupling-aware efficiency modeling of outphasing power amplifiers for wearable wireless power transfer

**DOI:** 10.1038/s41598-026-56108-1

**Published:** 2026-06-03

**Authors:** Nizar Brahim, Fawwaz Hazzazi, Giuseppina Monti, Luciano Tarricone, Hichem Taghouti, Abdelkader Mami

**Affiliations:** 1 Department of physics, UR-LAPER, University of Tunis El Manar, Tunis, 1068 Tunisia; 2 Department of Electrical Engineering, College of Engineering, Prince Sattam bin Abdulaziz University, Al- Kharj, 11942 Saudi Arabia; 3 Department of Engineering for Innovation, University of Salento, Lecce, 73100 Italy; 4 High Institute of Environmental Sciences and Technologies of Borj Cedria, University of Carthage, Carthage, Tunisia

**Keywords:** Outphasing power amplifiers, wireless power transfer, power combiners, Wilkinson combiner, Chireix combiner, system-level modeling, efficiency analysis, wearable devices, Energy science and technology, Engineering, Mathematics and computing

## Abstract

Outphasing power amplifiers (PAs) enable high efficiency under output back-off through constant-envelope operation, making them attractive for wireless power transfer (WPT) systems. However, in practical wearable scenarios, overall efficiency is strongly influenced by the interaction between the power combiner and the coupling-dependent impedance of the inductive link—an effect that has not been systematically characterized. In this work, we present a system-level analytical framework that jointly models outphasing PA operation, Wilkinson and Chireix combiner behavior, and coupling-dependent WPT load variations. The proposed approach enables explicit determination of optimal operating conditions as a function of the coupling coefficient. The analysis shows that for the Wilkinson combiner, the optimal outphasing angle is always θ = 0°, with efficiency primarily limited by wireless link coupling. In contrast, for the Chireix combiner, peak efficiency consistently occurs at the compensation angle θc, while its magnitude is strongly modulated by coupling conditions. These results translate into practical design insights: Chireix-based architectures with θc = 60°–70° can outperform Wilkinson combiners by up to 8% points at moderate coupling (k ≈ 0.2), whereas Wilkinson configurations offer greater robustness under weak or dynamically varying coupling (k < 0.15). Circuit-level harmonic balance simulations for both Wilkinson and Chireix combiners further confirm the analytical trends, demonstrating that the predicted efficiency behavior is physically realizable for both topologies. The proposed framework provides actionable guidelines for early-stage design and architecture selection in wearable WPT systems without requiring device-level or full electromagnetic simulations.

## Introduction

Wireless power transfer (WPT) has emerged as a key enabling technology for wearable electronics, biomedical implants, and low-power Internet-of-Things (IoT) devices, where physical connectors are impractical or undesirable^1–3^. In such applications, system efficiency directly impacts battery lifetime, thermal constraints, and operational reliability. Inductive and resonant coupling techniques are particularly attractive for short-range wearable WPT links due to their simplicity and relatively high efficiency; however, their performance is strongly affected by variations in coupling caused by misalignment, motion, and changes in distance between transmitter and receiver coils^4–6^. These effects are especially pronounced in wearable scenarios, where user movement introduces continuously varying load conditions^4–8^.

On the transmitter side, radio-frequency (RF) power amplifiers (PAs) often operate under significant output back-off due to adaptive power control or high peak-to-average power ratio (PAPR) signals^9^. Outphasing power amplifiers provide an effective solution for maintaining high efficiency under back-off operation by driving multiple branch amplifiers with constant-envelope signals and controlling the output power through phase modulation^10,11^. While this architecture preserves intrinsic PA efficiency, the overall performance of an outphasing transmitter is strongly influenced by the characteristics of the power combiner used to recombine the branch signals.

Classical power combiners such as the Wilkinson and Chireix topologies exhibit fundamentally different behaviors under outphasing operation. The Wilkinson combiner provides good isolation and robustness against load mismatches but dissipates out-of-phase power in an isolation resistor, leading to efficiency degradation at high back-off levels^12,13^. In contrast, the Chireix combiner employs reactive compensation to enhance efficiency near a specific back-off operating point, at the expense of increased sensitivity to load variations and frequency detuning^12,14–17^. Although these characteristics are well understood for fixed resistive loads, their implications in wireless power transfer systems where the effective load impedance is inherently reactive and coupling-dependent remain insufficiently explored.

Most existing studies on WPT systems focus primarily on coil design, electromagnetic optimization, or link-level efficiency, often assuming ideal RF sources or fixed transmitter behavior^18–24^. Conversely, analyses of outphasing PAs and power combiners typically consider static, resistive loads and do not account for the dynamic impedance variations introduced by inductive wireless links^25–27^. As a result, the interaction between PA back-off operation, combiner efficiency, and coupling-dependent WPT load modulation is frequently overlooked. This separation can lead to suboptimal transmitter architectures and inaccurate efficiency predictions, particularly in wearable applications where coupling conditions vary continuously.

To address this gap, this paper presents a system-level efficiency modeling framework for outphasing power amplifier transmitters driving inductive wireless power transfer links with variable coupling conditions. The proposed approach explicitly integrates three interacting subsystems: the outphasing PA branches, the power combiner (Wilkinson or Chireix), and the WPT link modeled using a lumped-element representation. Closed-form analytical expressions are employed to capture combiner behavior and reflected load impedance variations as functions of the outphasing angle and coupling coefficient. This unified formulation enables joint evaluation of PA efficiency, combiner losses, and wireless link efficiency within a single analytical framework.

MATLAB-based simulations are used to investigate end-to-end efficiency trends and design trade-offs under realistic wearable operating conditions. The analysis demonstrates that optimizing PA operation alone is insufficient when coupling-dependent load variations dominate system behavior. The results highlight the complementary roles of Wilkinson and Chireix combiners: Wilkinson architectures offer robust and predictable performance under varying load conditions, while Chireix-based solutions achieve higher efficiency near moderate back-off when sufficient coupling is available.

The main contributions of this work can be summarized as follows:

- System-level PA–combiner–WPT modeling: A unified analytical framework is developed that jointly captures outphasing PA operation, power combiner behavior, and coupling-dependent WPT load modulation.
- Efficiency analysis under dynamic coupling: The impact of wearable-induced coupling variations on end-to-end efficiency is quantified, revealing scenarios where conventional PA-level optimization is insufficient.
- Comparative combiner evaluation: The performance trade-offs between Wilkinson and Chireix combiners are analyzed in the context of inductive WPT links with variable coupling.
- Practical design insight: The proposed framework provides actionable guidance for early-stage architecture selection and efficiency optimization without requiring computationally intensive electromagnetic or transistor-level simulations. Circuit-level harmonic balance simulations for both combiner topologies provide independent validation of the analytical trends.

While individual models for outphasing PAs, Wilkinson/Chireix combiners, and inductive WPT links exist in the literature, no prior work has integrated them into a unified framework that explicitly captures the cross-dependence between outphasing angle, combiner behavior, and coupling-dependent reflected impedance. This integration is not merely organizational — it enables analytical results that are not readily available from isolated subsystem analysis. Specifically, it allows closed-form derivation of the optimal outphasing angle θ(k) for each combiner topology as a function of coupling coefficient, revealing that the Wilkinson optimum is always θ* = 0° while the Chireix optimum is always θ* = θc, but with efficiency magnitudes that are differently and strongly modulated by k. These results provide concrete, coupling-aware design guidelines that are directly actionable for wearable WPT system architects.

The remainder of this paper is organized as follows. Section II presents the system-level modeling framework for the outphasing PA, power combiners, and wireless power transfer link. Section III describes the simulation setup and parameter selection. Section IV discusses the simulation results, design insights, and circuit-level validation of the analytical framework. Section V provides a discussion of practical implications and limitations, and Section VI concludes the paper.

## System-level modeling framework

### Overall system description

**Fig. 1:**
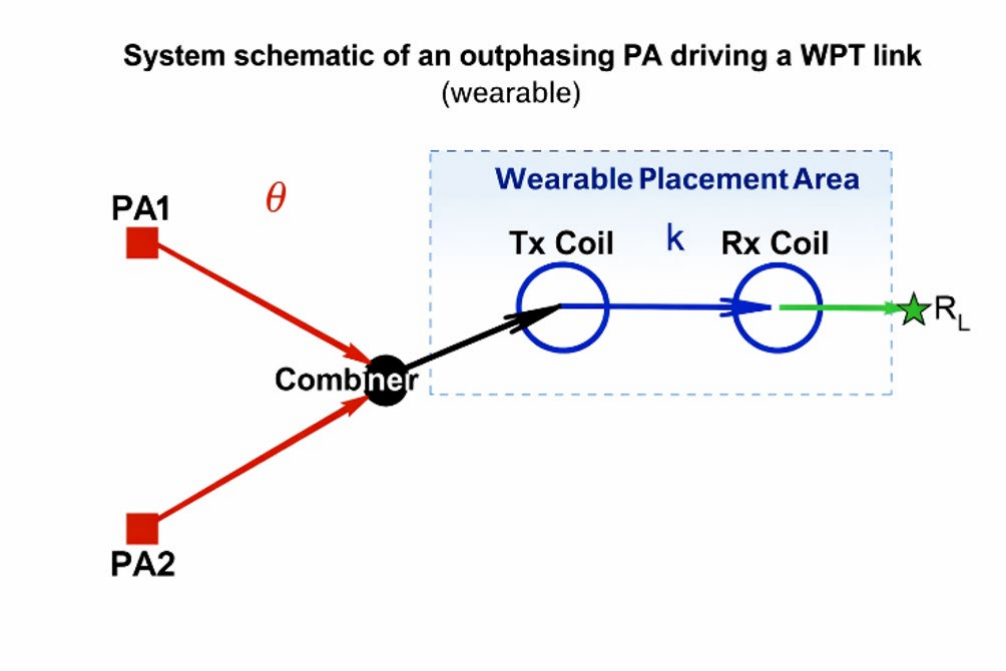
System-level schematic of an outphasing power amplifier driving an inductive wireless power transfer link. Two constant-envelope PA branches are combined using a power combiner and drive the transmitter coil. The effective load impedance varies with the coupling coefficient between the transmitter and receiver coils, which is representative of wearable operating conditions. The dashed rectangle indicates the wearable placement area, where coupling varies due to user motion and misalignment.

The considered system consists of an outphasing power amplifier (PA) transmitter driving an inductive wireless power transfer (WPT) link, as illustrated in Fig. 1.

The objective of the proposed modeling framework is to evaluate the end-to-end efficiency of the transmitter–link system under output back-off and coupling-dependent load variations, which are characteristic of wearable applications.

The transmitter employs two identical PA branches driven by constant-envelope signals of equal amplitude. Output power control is achieved by adjusting the phase difference between the two branches, defined as the outphasing angle θ. The branch outputs are recombined using a power combiner, which can be either an isolating Wilkinson topology or a non-isolating Chireix topology. The choice of combiner directly affects how out-of-phase power components are handled and, consequently, the efficiency of the transmitter under back-off operation.

The combined RF signal drives the transmitter coil of an inductive WPT link. Power is transferred wirelessly to a receiver coil through magnetic coupling and delivered to a resistive load at the receiver side. From the perspective of the transmitter, the WPT link presents a reflected load impedance that depends on the coupling coefficient between the coils. In wearable scenarios, this coupling coefficient varies dynamically due to user motion, coil misalignment, and changes in separation distance, resulting in a time-varying and generally reactive load at the PA output.

To capture the dominant efficiency trends while maintaining analytical transparency, the system is modeled at a first-order, system level. The PA branches are represented as ideal voltage sources operating under constant-envelope excitation, and their intrinsic efficiency is assumed to remain constant when properly loaded. Power combiner behavior is described using closed-form efficiency expressions that capture isolation losses in the Wilkinson topology and reactive compensation effects in the Chireix topology. The WPT link is modeled using a lumped-element representation that accounts for coupling-dependent reflected impedance.

This modeling approach intentionally avoids transistor-level nonlinearities and full-wave electromagnetic simulations in order to enable rapid parametric exploration of design trade-offs. Despite this abstraction, the framework preserves the essential interaction between outphasing control, power combiner behavior, and coupling-induced load modulation. As such, it provides meaningful insight into the relative contributions of the PA, combiner, and wireless link to the overall system efficiency, which is particularly valuable during early-stage architecture selection and co-design of outphasing WPT transmitters.

### Outphasing power amplifier model

Outphasing power amplifiers achieve output power control by exploiting phase modulation rather than amplitude modulation. In this architecture, two parallel PA branches are driven by constant-envelope signals of equal amplitude, while the output power is adjusted by varying the phase difference between the branch signals, commonly referred to as the outphasing angle θ^10,11^. This operating principle enables high intrinsic efficiency under output back-off, making outphasing architectures attractive for applications such as wearable wireless power transfer (WPT), where power levels and loading conditions vary dynamically.

**Fig. 2:**
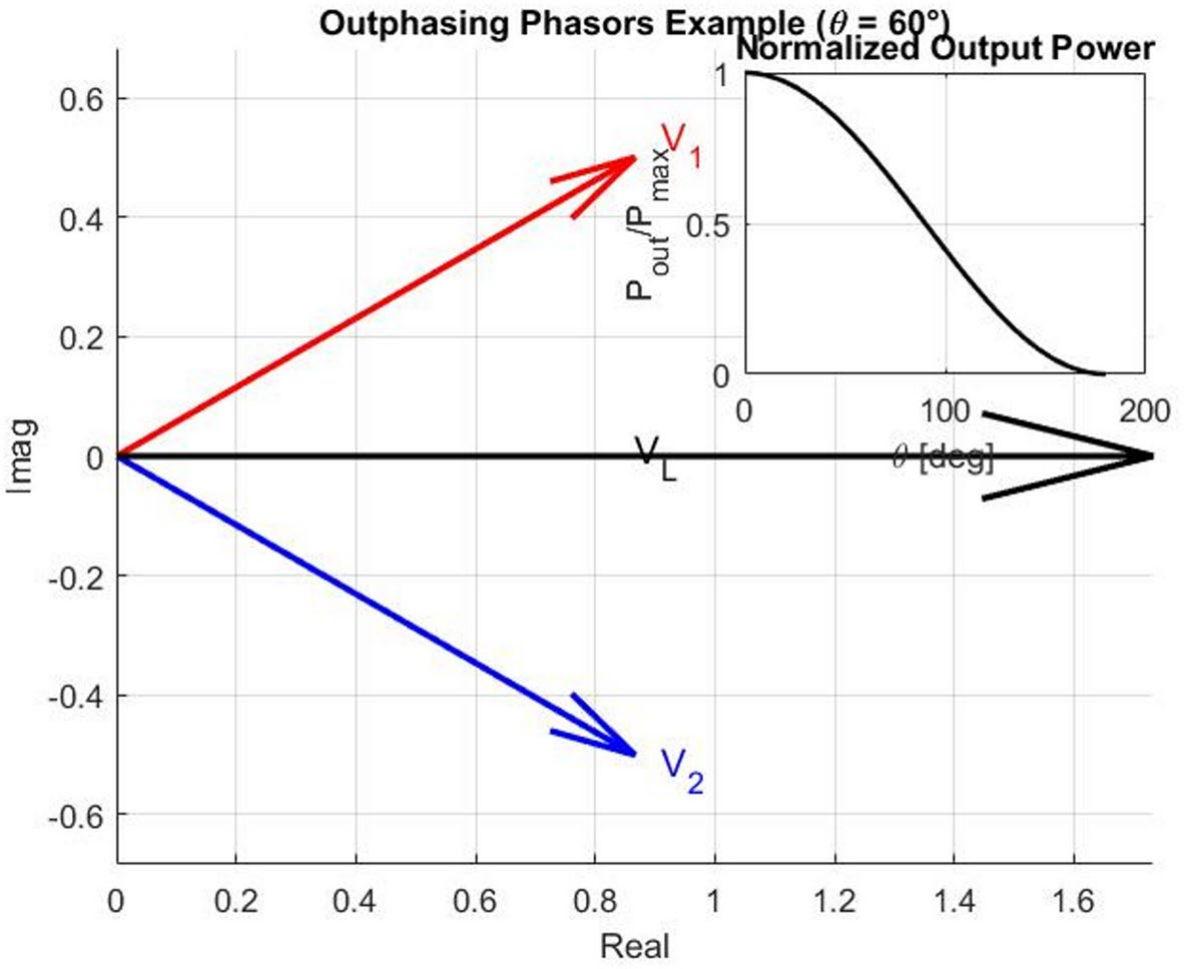
Phasor representation of an outphasing power amplifier. Two constant-amplitude branch voltages are combined with a phase difference θ, producing an output voltage whose magnitude depends on the outphasing angle.

Figure 2 provides a phasor representation of the outphasing operation and visually illustrates how the output voltage is formed by vector addition of the two branch voltages. Introducing the figure at this point allows the phase-based power control mechanism to be understood intuitively before presenting the analytical expressions.

As illustrated in Fig. 2, each PA branch delivers a sinusoidal voltage of constant magnitude V_0_. In phasor notation, the branch voltages can be expressed as:

1 $$\:{V}_{1}={V}_{0}.{e}^{j\frac{\theta\:}{2}}$$

2 $$\:{\:\:\:\:\:\:\:\:\:V}_{2}={V}_{0}.{e}^{-j\frac{\theta\:}{2}}\:\:\:\:\:\:\:$$

Where θ denotes the outphasing angle between the two branches. The combined load voltage V_L_ is obtained by vector summation of the branch voltages, resulting in a magnitude given by:

3 $$\:\left|{V}_{L}\right|=2{V}_{0}\mathrm{cos}\left(\frac{\theta\:}{2}\right)$$

This expression highlights the fundamental mechanism of outphasing operation: the output voltage, and hence the delivered power, decreases smoothly as the outphasing angle increases, while the branch voltage amplitudes remain constant.

Assuming a resistive reference load, the normalized output power follows the classical outphasing relationship^10,14,28,29^:

4 $$\:\frac{{P}_{out}}{{P}_{max}}={\mathrm{cos}}^{2}\left(\frac{\theta\:}{2}\right)$$

Where Pmax corresponds to the maximum output power achieved when the two branch signals are in phase (θ = 0). This relationship provides a convenient and widely accepted analytical description of output back-off behavior in outphasing transmitters.

In this work, the PA branches are modeled as ideal constant-envelope voltage sources with fixed intrinsic efficiency when properly loaded. As a result, variations in system efficiency under back-off are not attributed to changes in device-level PA performance, but rather to the behavior of the power combiner and the effective load impedance presented by the wireless power transfer link. This modeling choice is intentional and consistent with the system-level focus of the proposed framework, allowing the dominant interactions between outphasing control, power combining, and coupling-dependent load modulation to be isolated and analyzed without introducing unnecessary transistor-level complexity.

### Power combiner models

In outphasing transmitters, the power combiner plays a central role in determining how efficiently the branch amplifier outputs are recombined, particularly under output back-off operation. Although the branch PAs operate with constant-envelope excitation, the phase difference between their outputs causes a portion of the delivered power to circulate within the combining network. The manner in which this out-of-phase power is handled strongly influences the overall transmitter efficiency. In this work, two classical and widely used power combiner topologies are considered: the isolating Wilkinson combiner and the non-isolating Chireix combiner.

#### Wilkinson combiner

The Wilkinson combiner is an isolating network that provides good port matching and high isolation between the branch amplifiers under in-phase operation. When the two branch signals are phase-aligned, the isolation resistor does not dissipate power, and nearly all the supplied power is delivered to the load. As the outphasing angle θ increases, however, a growing fraction of the out-of-phase power component is diverted to the isolation resistor and dissipated as heat.

At a system level, the efficiency behavior of an ideal Wilkinson combiner under outphasing operation can be captured using a simple and well-established analytical expression^12,13^:

5 $$\:{\eta\:}_{\left\{comb,W\right\}\left(\theta\:\right)}=\:co{s}^{2\:}\left(\frac{\theta\:}{2}\right)$$

This expression directly mirrors the normalized output power relationship of the outphasing PA and reflects the fact that power associated with the quadrature component between the branch signals is lost in the isolation resistor. While this results in a monotonic efficiency degradation as back-off increases, the Wilkinson combiner offers strong robustness against load mismatches and variations at the output port.

This robustness is particularly relevant in wireless power transfer systems, where the effective load impedance seen by the transmitter varies with coupling conditions. The isolating nature of the Wilkinson combiner mitigates the impact of these variations on the branch amplifiers, making it a reliable choice for applications characterized by dynamic or poorly controlled loads, such as wearable WPT scenarios.

#### Chireix combiner

The Chireix combiner represents a non-isolating alternative designed to improve efficiency under output back-off by exploiting reactive load modulation. In this architecture, reactive compensation elements of opposite sign are connected at the outputs of the branch amplifiers. These elements are selected such that, at a specific outphasing angle known as the compensation angle θc, the effective branch loads become predominantly resistive.

Near this compensation point, the circulation of reactive power within the combining network is minimized, leading to a significant improvement in combiner efficiency compared to isolating topologies^12,14–16^. Away from θc, residual reactive components reappear, and efficiency gradually degrades as the operating point moves further from the compensated condition.

To capture this behavior within a system-level modeling framework, the efficiency of the Chireix combiner is represented using a phenomenological expression that describes the efficiency envelope around the compensation angle:

6 $$\:{\eta\:}_{\left\{comb,\:\:C\right\}\left(\theta\:\right)}\:=\:{e}^{-\alpha\:{(\theta\:\:-\:{\theta\:}_{c})}^{2}}$$

Where α is a tuning parameter that controls the sensitivity of the combiner efficiency to deviations from θc. This representation does not aim to reproduce detailed circuit-level behavior, but rather to model the dominant efficiency trends observed in compensated outphasing combiners. Similar abstraction levels have been adopted in prior system-level studies to enable efficient exploration of architectural trade-offs without resorting to harmonic-balance or transistor-level simulations^25–27^.

While the Chireix combiner can achieve higher efficiency than the Wilkinson topology at moderate back-off levels, its performance is inherently more sensitive to load impedance variations and operating frequency. In wireless power transfer applications, where the effective load is coupling-dependent and often reactive, this sensitivity introduces a trade-off between peak efficiency and robustness. As a result, the benefits of reactive compensation are most pronounced when the WPT link exhibits sufficiently strong and stable coupling conditions.

### Wireless power transfer link model

The wireless power transfer (WPT) system is modeled using a lumped-element representation of an inductively coupled link, which is widely adopted for analyzing short-range WPT systems operating in the near field^4–6,18,21^. As illustrated in Fig. 1, the transmitter coil with inductance L_tx_ is magnetically coupled to a receiver coil with inductance L_rx_, which delivers power to a resistive load at the receiver side. This modeling approach captures the dominant electrical behavior of the WPT link while remaining analytically tractable and suitable for system-level efficiency analysis.

The magnetic coupling between the transmitter and receiver coils is characterized by the mutual inductance M, which can be expressed as

7 $$\:M\:=\:k\:\sqrt{{L}_{tx}{L}_{rx}}$$

Where k is the coupling coefficient. In wearable applications, the coupling coefficient is not constant and varies due to changes in coil alignment, separation distance, and relative orientation caused by user motion^4^–^6,22,30^. As a result, the electrical load presented to the transmitter is inherently dynamic and depends on the operating conditions of the WPT link.

From the perspective of the transmitter, the receiver circuit reflects an equivalent impedance back to the transmitter coil. Assuming sinusoidal steady-state operation at angular frequency ω, the reflected impedance can be expressed as^18,19,21,23^

8 $$\:{Z}_{ref}=\frac{\left({\omega\:}^{2}{M}^{2}\right)}{\left({R}_{rx}+\:j\:\omega\:\:{L}_{rx}\right)}$$

Where R_rx_ represents the effective resistive load at the receiver side. The reflected impedance is generally complex-valued and varies with the coupling coefficient k, introducing both resistive and reactive components into the load seen by the transmitter. The wireless power transfer efficiency ηWPT is then obtained from this reflected impedance using the standard inductive link formula:

$$\:{\eta\:}_{WPT}\:=\frac{{\omega\:}^{2}{M}^{2}{R}_{L}}{\left(\left({R}_{tx}+\:Re\left({Z}_{ref}\right)\right)\left({R}_{L}+\:{R}_{rx}\right)\right)}$$^18,21^.

This expression is used throughout the simulations to compute the link contribution as a function of k.

Consequently, the effective load impedance presented to the outphasing power amplifier is not fixed, but instead depends on the wireless link conditions. In wearable scenarios, where coupling variations occur continuously during normal operation, this coupling-dependent reflected impedance introduces an additional source of load modulation on top of that caused by outphasing operation. This interaction directly influences the behavior of the power combiner and the overall system efficiency, particularly for non-isolating architectures that are sensitive to reactive loading.

The adopted lumped-element WPT model does not aim to capture detailed electromagnetic field distributions or coil geometry optimization. Rather, it is intended to provide a physically meaningful and computationally efficient representation of coupling-induced load variations for system-level analysis. This abstraction is well suited to the objectives of this work, which focus on evaluating efficiency trends and design trade-offs arising from the interaction between outphasing control, power combiner behavior, and wireless link dynamics under realistic wearable operating conditions.

## Simulation setup and parameter selection

### End-to-end efficiency definition

To evaluate the performance of the outphasing power amplifier transmitter driving a wireless power transfer (WPT) link, an end-to-end efficiency metric is adopted. This metric captures the combined impact of the power amplifier operation, power combiner behavior, and wireless link characteristics, enabling a unified assessment of system performance under output back-off and coupling-dependent load variations.

The total system efficiency is defined as the product of three contributing efficiency terms:

9 $$\:{\eta\:}_{total}={\eta\:}_{PA}.{\eta\:}_{comb}.{\eta\:}_{WPT}\:$$

where ηPA represents the intrinsic efficiency of the PA branches, ηcomb denotes the efficiency of the power combiner, and ηWPT corresponds to the efficiency of the wireless power transfer link.

For clarity, the separability of efficiency terms is summarized below:

**Table:** 

Quantity	Formula	Depends on
η_PA	constant	—
η_comb	Equation (5) or (6)	θ only
η_WPT	Equations (7)–(8)	k only
η_total	η_PA × η_comb × η_WPT	θ and k

This separability means that efficiency trends under outphasing control (θ) and coupling variations (k) can be analyzed independently and combined multiplicatively, enabling transparent identification of the dominant loss mechanism at each operating point.

The PA efficiency term ηPA reflects the constant-envelope operation of the outphasing architecture. In this work, the PA branches are assumed to operate with fixed intrinsic efficiency when properly loaded, and therefore ηPA is treated as constant across the considered outphasing range. This assumption allows the analysis to focus on efficiency variations arising from power combining losses and wireless link dynamics, which are the dominant factors in wearable WPT scenarios. The combiner efficiency term ηcomb accounts for losses associated with recombining the branch signals under outphasing operation. As discussed in Section II.C, this term depends explicitly on the outphasing angle and the selected combiner topology. For isolating combiners such as the Wilkinson architecture, efficiency degradation is primarily caused by dissipation of out-of-phase power in the isolation resistor. For non-isolating combiners such as the Chireix topology, efficiency is governed by reactive compensation effects and exhibits a peak near the compensation angle.

The wireless power transfer efficiency ηWPT captures losses in the inductive link between the transmitter and receiver coils. This term depends on the coupling coefficient and the reflected load impedance seen by the transmitter, as described in Section II.D. In wearable applications, variations in coupling due to motion and misalignment directly influence ηWPT, making it a key determinant of the overall system efficiency.

By expressing the total efficiency as the product of these three contributions, the proposed framework enables clear identification of dominant loss mechanisms while preserving the interaction between the transmitter and the wireless link. This formulation is particularly well suited for system-level analysis and parametric exploration, as it allows efficiency trends to be evaluated as functions of the outphasing angle, combiner topology, and coupling conditions without resorting to computationally intensive circuit- or field-level simulations.

### Simulation parameters and assumptions

The proposed system-level efficiency analysis is carried out using a MATLAB-based simulation framework that combines the analytical models introduced in Section II. The objective of the simulations is to evaluate relative efficiency trends and design trade-offs of outphasing power amplifier transmitters driving inductive wireless power transfer (WPT) links under output back-off and coupling-dependent load variations. The selected parameters are representative of short-range wearable WPT systems and are chosen to balance physical realism with analytical simplicity.

The main simulation parameters used throughout this study are summarized in Table 1. These parameters define the operating frequency, inductive link characteristics, outphasing control range, and combiner-related settings employed in the analysis.

**Table 1:** key simulation parameters used in the system-level analysis.

Parameter	Symbol	Value / Range	Notes
Operating frequency	( f )	13.56 MHz	ISM band, typical for WPT
Transmitter coil inductance	( Ltx )	10 µH	Wearable-scale inductive link
Receiver coil inductance	( Lrx )	10 µH	Wearable-scale inductive link
Receiver load resistance	( Rrx )	50 Ω	Reference resistive load
Coupling coefficient	( k )	0.1–0.3	Typical for dynamic body-worn inductive links
Outphasing angle sweep	( θ )	0°–120°	Output back-off control
Chireix compensation angle	( θ_C_ )	60°–80°	Corresponding to 6–10 dB back-off
Branch voltage amplitude	( V_0_ )	1 (normalized)	Constant-envelope excitation
Chireix tuning parameter	( α )	Selected for peak at (θ_C_)	System-level abstraction

The operating frequency is set to 13.56 MHz, which lies within the industrial, scientific, and medical (ISM) band and is commonly used for near-field wireless power transfer applications^3,30^. The transmitter and receiver inductances are selected to reflect typical wearable-scale coils reported in the literature^4–6,22^. The coupling coefficient is varied over a range representative of realistic wearable scenarios, encompassing coil misalignment, motion, and moderate changes in separation distance.

Several modeling assumptions are adopted to maintain analytical transparency and focus on dominant system-level interactions. The receiver load resistance Rrx is fixed at 50 Ω, representing the equivalent resistive termination after the receiver rectifier in typical wearable WPT systems^18,21^.

The PA branches are assumed to operate with constant intrinsic efficiency due to constant-envelope excitation, and losses associated with matching networks and active device nonidealities are neglected. This allows efficiency variations to be attributed primarily to power combiner behavior and coupling-dependent wireless link effects. For the Chireix combiner, the compensation angle θc and tuning parameter α are selected to reproduce typical efficiency envelopes observed in compensated outphasing architectures^12,14,15^.

These assumptions are consistent with the objectives of the proposed framework, which aims to provide comparative insight into architectural trade-offs and efficiency trends rather than absolute performance prediction. By clearly defining simulation parameters and assumptions, the analysis remains reproducible and well suited for early-stage design exploration of outphasing WPT transmitters under realistic wearable operating conditions.

### Simulation procedure

The end-to-end efficiency analysis is performed using a deterministic MATLAB-based simulation workflow that integrates the analytical models introduced in Sections II and III.A. The simulation procedure is designed to enable systematic and reproducible evaluation of efficiency trends as functions of the outphasing angle, power combiner topology, and wireless power transfer (WPT) coupling conditions. No numerical optimization or iterative solvers are employed; instead, parametric sweeps are used to clearly isolate the impact of each design variable.

The simulation procedure consists of the following steps:

Step 1: Outphasing Control Sweep.

The outphasing angle θ is swept over the range defined in Table 1, from in-phase operation (θ = 0∘) to deep back-off conditions. For each value of θ, the branch voltages are computed according to the outphasing PA model described in Section II.B, and the combined output voltage is obtained through vector summation. The corresponding normalized output power is then evaluated using the classical outphasing relationship.

Step 2: Power Combiner Efficiency Application.

For each outphasing angle, the efficiency of the selected power combiner is applied to the ideal output power. Two cases are considered: the Wilkinson combiner and the Chireix combiner. The combiner efficiency is computed using the analytical expressions introduced in Section II.C, allowing the influence of isolation losses or reactive compensation effects to be captured as a function of θ.

Step 3: Wireless Power Transfer Link Modeling.

The WPT link is modeled by selecting a value of the coupling coefficient k within the specified range and computing the corresponding mutual inductance. Using the lumped-element formulation described in Section II.D, the reflected impedance presented to the transmitter is evaluated for each coupling condition. This step introduces coupling-dependent load modulation into the simulation framework.

Step 4: Wireless Link Efficiency Evaluation.

Based on the reflected impedance and receiver load parameters, the WPT efficiency ηWPT is computed for each value of the coupling coefficient. This efficiency term captures losses associated with the inductive link and reflects the impact of wearable-induced variations such as misalignment and distance changes.

Step 5: End-to-End Efficiency Computation.

The total system efficiency is calculated by combining the individual efficiency contributions according to the definition provided in Section III.A. For each combination of outphasing angle, combiner topology, and coupling coefficient, the end-to-end efficiency is obtained as the product of the PA, combiner, and WPT efficiency terms. The plotted results in Section IV are generated directly from the analytical expressions in Sections II.B–II.D and the end-to-end efficiency definition in (9), with no additional fitting or empirical adjustment.

Step 6: Parametric Repetition and Data Collection.

The above steps are repeated for all considered values of the coupling coefficient and for both power combiner topologies. The resulting efficiency data are stored and organized for post-processing and visualization, enabling direct comparison of efficiency trends across different operating conditions.

This structured simulation procedure ensures a clear and consistent coupling between outphasing control, power combining behavior, and wireless link dynamics. By relying on analytical expressions and parametric sweeps, the framework enables efficient exploration of design trade-offs and provides transparent insight into the factors that dominate end-to-end efficiency in wearable outphasing WPT transmitters.

## Simulation results and discussion

### End-to-end efficiency versus outphasing angle

The end-to-end efficiency of the outphasing power amplifier transmitter is first evaluated as a function of the outphasing angle θ, which directly controls the output back-off level. This analysis provides a global view of system behavior and highlights the influence of power combiner topology under realistic wireless power transfer (WPT) operating conditions.

**Fig. 3:**
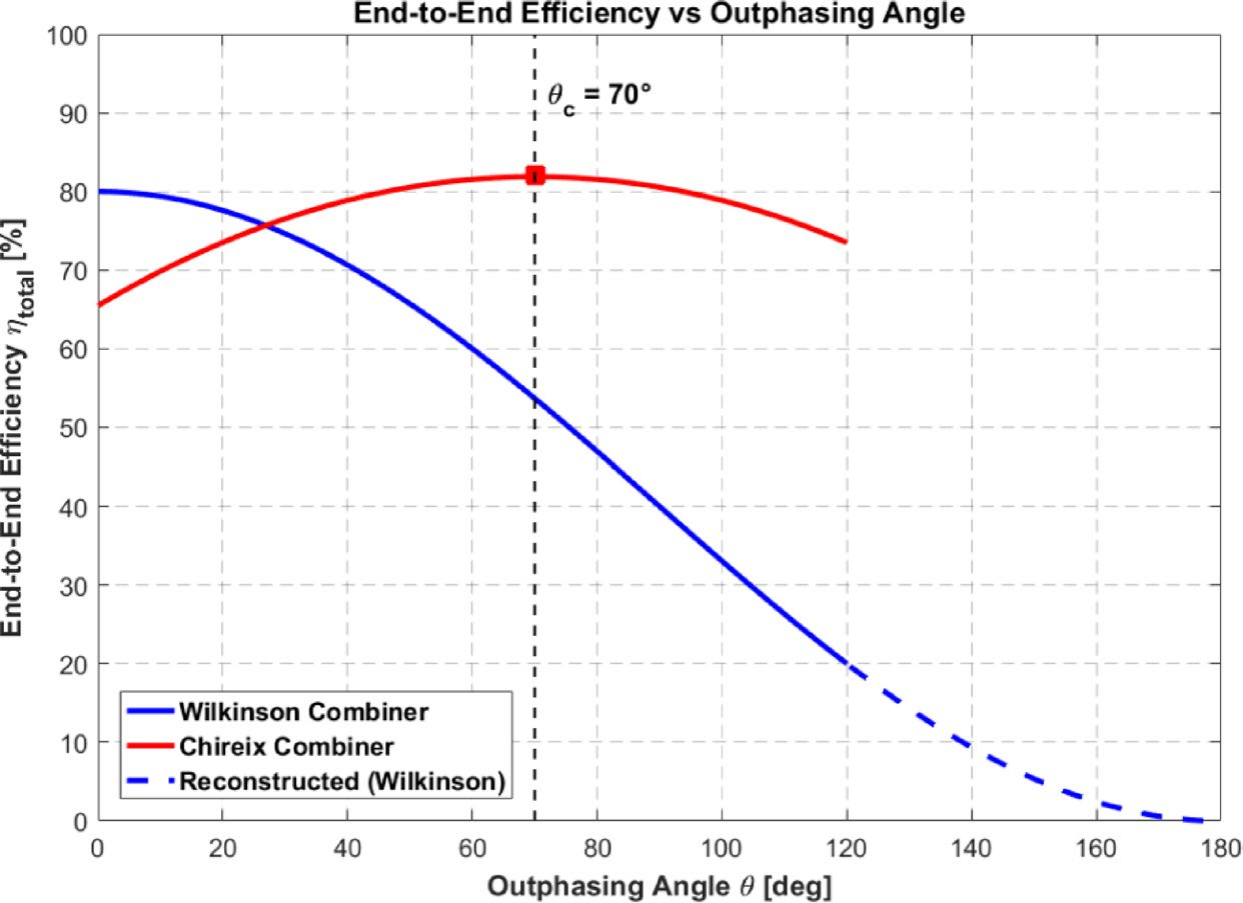
End-to-end efficiency η_total versus outphasing angle θ for Wilkinson and Chireix combiners under an ideal wireless link (η_WPT = 1.0, η_PA = 0.80). The dashed blue line shows the reconstructed efficiency from η_PA × η_comb(θ) × η_WPT, which perfectly overlaps the solid Wilkinson curve from 0° to 120° (demonstrating consistency) and continues to 180° to show the full theoretical trend. The red square marks the Chireix peak at θ_c = 70°.

Figure 3 illustrates the total system efficiency ηtotal as a function of the outphasing angle for both Wilkinson and Chireix power combiners, using the efficiency definition and simulation procedure described in Section III. Presenting this figure at the beginning of the results section allows the dominant efficiency trends to be observed before examining individual loss mechanisms. Unless otherwise stated, the results in Fig. 3 are obtained for a representative coupling coefficient k = 0.2, corresponding to moderate wearable coupling.

As shown in Fig. 3, the Wilkinson combiner exhibits a smooth and monotonic decrease in end-to-end efficiency as the outphasing angle increases. This behavior is consistent with the isolating nature of the Wilkinson topology, where out-of-phase power components are increasingly dissipated in the isolation resistor as back-off increases. While this results in reduced efficiency at large outphasing angles, the efficiency trend remains predictable and stable across the entire operating range.

In contrast, the Chireix combiner demonstrates a distinct efficiency peak near the compensation angle θc, corresponding to moderate output back-off. In this region, reactive compensation effectively minimizes circulating reactive power within the combining network, leading to improved efficiency compared to the Wilkinson topology. Beyond the compensated operating point, the efficiency gradually degrades as the operating condition moves away from optimal reactive balance. This characteristic behavior is consistent with previously reported analyses of compensated outphasing architectures and load-modulated power amplifiers^25–27^.

The comparison in Fig. 3 highlights a fundamental design trade-off between robustness and peak efficiency. The Wilkinson combiner provides reliable performance over a wide outphasing range and is less sensitive to load variations, making it attractive for operating conditions characterized by uncertainty or dynamic loading. The Chireix combiner, on the other hand, offers superior efficiency near a targeted back-off region but requires careful selection of the compensation angle and sufficiently favorable load conditions to fully realize its benefits. Similar trade-offs have been reported in recent reviews of high-efficiency outphasing and load-modulated power amplifier architectures^14,25^. To provide a more practical metric, the average end-to-end efficiency over the full outphasing range (θ = 0° to 120°) at k = 0.2 is approximately 62% for the Wilkinson combiner and 68% for the Chireix combiner. When averaged only over moderate back-off (θ = 40°–80°), the Chireix combiner reaches 72%, outperforming the Wilkinson by about 8% points under favorable coupling.

It is important to note that the observed efficiency trends reflect relative system-level behavior rather than absolute performance values. The results emphasize how power combiner selection and outphasing control jointly shape end-to-end efficiency, even before considering variations in wireless coupling. This observation motivates the subsequent analysis, which decomposes efficiency contributions and examines the impact of coupling-dependent WPT load variations in greater detail.

### Efficiency breakdown of system components

Because η_PA and η_WPT are both independent of θ for fixed coupling conditions, the variation of η_total with outphasing angle follows directly from η_comb(θ) alone. This multiplicative separability can be verified numerically using the analytical expressions. For example, at θ = 60° in the Wilkinson case, Eq. (5) gives η_comb = cos²(30°) = 0.75. With η_PA = 0.80 and η_WPT = 1.0 (the value used in Fig. 3), the product 0.80 × 0.75 × 1.0 = 0.60, which matches the corresponding η_total ≈ 0.60 in Fig. 3.

To gain deeper insight into the efficiency trends observed in Fig. 3, the end-to-end efficiency is decomposed into its constituent contributions: the power amplifier (PA) branches, the power combiner, and the wireless power transfer (WPT) link. This breakdown allows the dominant loss mechanisms to be identified across the outphasing operating range and clarifies the respective roles of the transmitter architecture and the wireless link.

**Fig. 4:**
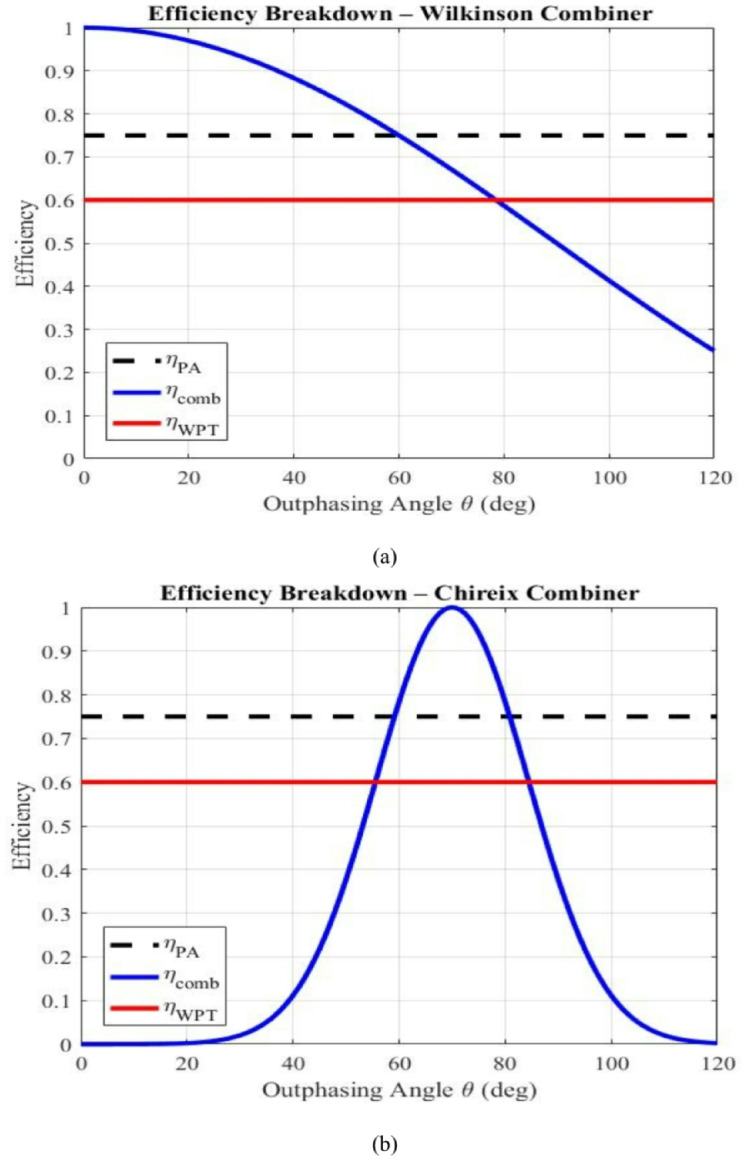
Efficiency breakdown versus outphasing angle θ for (**a**) Wilkinson and (**b**) Chireix combiners at k = 0.2. The PA efficiency is constant (η_PA = 0.80), the combiner efficiency follows Eqs. (5) and (6), and η_WPT is fixed at unity. The dashed line in Fig. 3 already provides visual verification of decomposition consistency, as described in its caption. (**a**) Wilkinson, (**b**) Chireix.

Figure 4 presents the individual efficiency contributions ηPA, ηcomb, and ηWPT as functions of the outphasing angle for both Wilkinson and Chireix combiner configurations. Introducing this figure immediately after the global efficiency trends enables direct correlation between subsystem behavior and the overall system performance.

As expected from the constant-envelope assumption adopted in Section II.B, the PA efficiency contribution remains constant across the entire outphasing range. This confirms that variations in end-to-end efficiency are not driven by intrinsic PA behavior, but instead arise from power combining losses and wireless link effects. This observation reinforces the relevance of system-level analysis when evaluating outphasing transmitters for wearable WPT applications.

For the Wilkinson combiner, Fig. 4 shows that efficiency degradation with increasing outphasing angle is dominated by losses in the combining stage. As the phase difference between branch signals increases, a growing portion of the delivered power is dissipated in the isolation resistor, leading to a monotonic reduction in ηcomb. The WPT efficiency contribution remains relatively stable for a given coupling condition, indicating that the wireless link does not compensate for combiner-induced losses under back-off operation.

In contrast, the Chireix combiner exhibits a pronounced efficiency enhancement near the compensation angle θc, where reactive load modulation minimizes circulating power within the combining network. In this region, the combiner contribution approaches its maximum value, resulting in a noticeable improvement in total system efficiency. Away from the compensated operating point, residual reactive effects cause the combiner efficiency to decrease, consistent with the trends observed in Fig. 3 and with reported behavior in compensated outphasing architectures^14,26,27^.

Across both combiner configurations, Fig. 4 also highlights the constraining role of the wireless power transfer link. Even when the PA and combiner operate near their optimal efficiency, the WPT contribution limits the achievable end-to-end efficiency, particularly under conditions of moderate or weak coupling. This result underscores that transmitter-side optimization alone is insufficient in wearable WPT systems and that wireless link characteristics must be considered jointly with PA and combiner design.

Overall, the efficiency breakdown confirms that the dominant loss mechanisms shift with operating conditions: combiner losses govern performance under back-off, while WPT losses impose an upper bound on achievable efficiency. These insights provide a clear physical explanation for the trends observed in Section IV.A and motivate the subsequent analysis of coupling-dependent effects.

### Effect of coupling coefficient on end-to-end efficiency

**Fig. 5:**
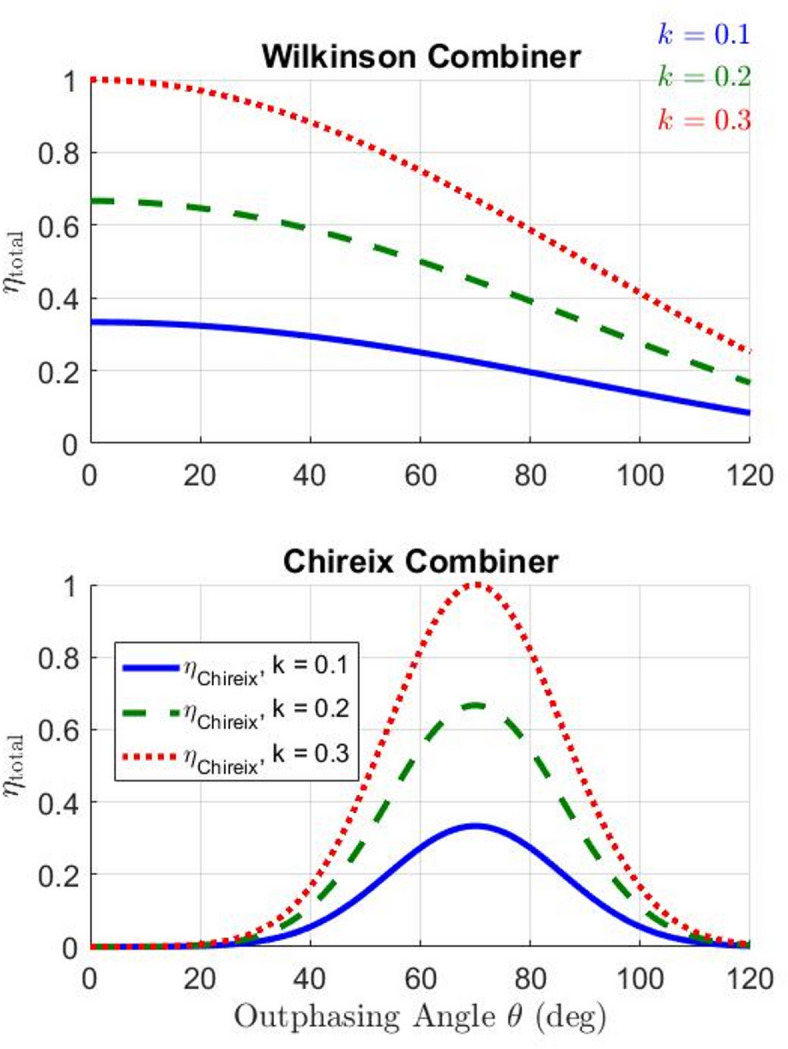
End-to-end efficiency versus outphasing angle for different wireless power transfer coupling coefficients and power combiner topologies.

In wearable wireless power transfer (WPT) applications, the coupling coefficient between the transmitter and receiver coils is rarely constant. Variations in alignment, distance, and orientation caused by user motion lead to time-varying coupling conditions, which can significantly influence the effective load impedance seen by the transmitter. To evaluate the impact of these variations, the end-to-end efficiency is analyzed for different values of the coupling coefficient k, while maintaining the same outphasing control and simulation parameters described in Section III. Figure 5 extends the results of Fig. 3 by sweeping the coupling coefficient k, where Fig. 3 corresponds to the single slice k = 0.2.

As shown in Fig. 5, reduced coupling coefficients lead to a substantial degradation in end-to-end efficiency across the entire outphasing range, regardless of the selected power combiner topology. Under weak coupling conditions, the wireless link efficiency dominates the overall performance, effectively limiting the amount of power that can be delivered to the receiver even when the transmitter operates near its optimal efficiency. This behavior reflects a fundamental constraint of inductive WPT systems and has been widely reported in studies addressing wearable and implantable power transfer^3,22,24,31^.

For moderate coupling values, differences between combiner topologies become more pronounced. In this regime, the Chireix combiner achieves higher efficiency near the compensation angle θc, benefiting from reactive load modulation when the wireless link is sufficiently strong. The Wilkinson combiner, while exhibiting lower peak efficiency, maintains a smoother and more predictable efficiency profile across the outphasing range. This robustness can be advantageous in wearable environments where coupling conditions fluctuate rapidly and precise compensation is difficult to maintain.

At higher coupling coefficients, corresponding to favorable alignment and short separation distances, both combiners approach near-ideal performance at low outphasing angles. However, the Chireix topology continues to offer superior efficiency at moderate back-off, confirming its suitability for scenarios involving high peak-to-average power ratios when coupling conditions are relatively stable. Similar observations have been reported in recent system-level analyses of WPT transmitters and load-modulated power amplifier architectures^27,32^.

The results in Fig. 5 highlight a critical system-level insight: transmitter-side efficiency optimization cannot compensate for weak wireless coupling. In wearable applications, where coupling variations are inevitable, the benefits of advanced combiner architectures are constrained by the wireless link itself. Consequently, effective system design requires joint consideration of PA back-off strategy, power combiner selection, and coil placement to ensure acceptable efficiency across realistic operating conditions. Recent studies on wearable WPT systems similarly emphasize the dominant role of coupling robustness over isolated transmitter optimization^22,23^.

Overall, this analysis demonstrates that the choice between Wilkinson and Chireix combiners should be guided not only by desired back-off efficiency, but also by expected coupling stability. These findings reinforce the importance of holistic PA–combiner–WPT co-design for achieving reliable and efficient power delivery in wearable wireless systems.

### Joint influence of outphasing angle and coupling coefficient

To provide a comprehensive view of the interaction between transmitter control and wireless link conditions, the end-to-end efficiency is analyzed as a joint function of the outphasing angle θ and the coupling coefficient k. This two-dimensional representation enables visualization of the full operating space encountered in wearable wireless power transfer (WPT) systems, where both transmitter back-off and coupling variations occur simultaneously.

**Fig. 6:**
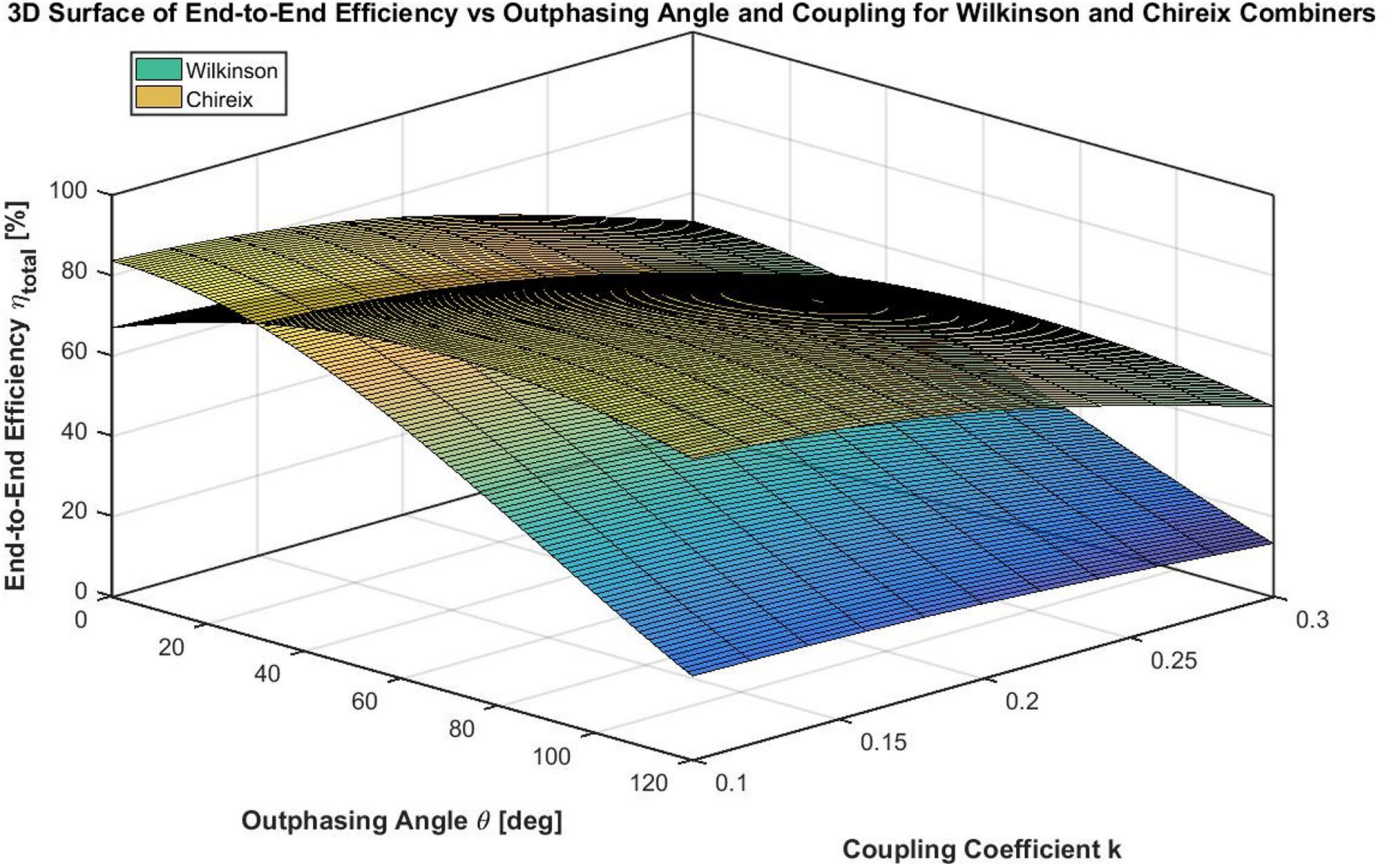
Three-dimensional surface of end-to-end efficiency as a function of outphasing angle and coupling coefficient for Wilkinson and Chireix power combiners.

Figure 6 presents a three-dimensional surface plot of the end-to-end efficiency as a function of the outphasing angle and coupling coefficient for the considered power combiner topologies. Introducing this figure at this stage provides a global perspective that complements the one-dimensional analyses presented in Sections IV.A–IV.C.

As illustrated in Fig. 6, the Wilkinson combiner exhibits a smooth and monotonically decreasing efficiency surface with increasing outphasing angle, largely independent of coupling variations. This behavior reflects the inherent robustness of isolating architectures, where efficiency degradation is primarily governed by dissipative losses in the isolation resistor rather than by sensitivity to load reactance. Consequently, the Wilkinson topology provides predictable performance across a wide range of operating conditions, which can be advantageous in wearable scenarios characterized by rapid and uncontrolled coupling fluctuations.

In contrast, the Chireix combiner displays a pronounced efficiency ridge near the compensation angle θc, particularly at higher coupling coefficients. This ridge corresponds to operating regions where reactive load compensation is effective and circulating power within the combiner is minimized. As coupling weakens or the operating point deviates from the compensation condition, the efficiency surface declines more sharply than in the Wilkinson case, highlighting the increased sensitivity of non-isolating combiners to coupling-dependent load variations.

The joint analysis in Fig. 6 reveals distinct operating regions that are relevant for system-level design. At low coupling coefficients, the efficiency surface is compressed for both combiners, indicating that wireless link limitations dominate overall performance regardless of transmitter architecture. At moderate to high coupling levels, however, the choice of power combiner and the selection of the outphasing operating range significantly influence achievable efficiency. In these regions, Chireix-based solutions offer higher efficiency at moderate back-off, while Wilkinson-based solutions maintain broader robustness.

This global visualization reinforces the key insight of this work: optimal performance in wearable outphasing WPT systems cannot be achieved by optimizing individual subsystems in isolation. Instead, transmitter control, power combiner topology, and wireless coupling conditions must be jointly considered. The three-dimensional efficiency surfaces provide designers with a practical tool for identifying operating regions that balance efficiency, robustness, and sensitivity to coupling variations, supporting informed architecture selection during early-stage system design.

### Optimal operating conditions as a function of coupling

The analytical framework presented in Section II enables systematic derivation of the optimal outphasing angle θ*(k) that maximizes end-to-end efficiency for a given coupling condition. Since η_PA is constant and η_WPT depends only on k and not on θ, maximizing η_total with respect to θ reduces to maximizing η_comb. For the Wilkinson combiner, the efficiency is given by:

$$\eta _{{comb,W}} (\theta )\, = \,\cos (\theta /2)$$

which is strictly decreasing over the considered range of θ. Therefore, the optimal operating point is:

$$\theta {*_W}={\text{ }}0^\circ \forall k$$

The corresponding maximum end-to-end efficiency is:

$$\eta {*_{total,W}}\left( k \right){\text{ }}=\eta \_PA{\text{ }}\cdot\eta \_WPT\left( k \right),$$

which depends solely on the coupling coefficient through η_WPT(k).

For the Chireix combiner, the efficiency is modeled as:

$$\eta _{{comb,c}} (\theta )\, = \,\exp ( - \alpha (\theta {-}\theta _{C} )^{2} )$$

which reaches its maximum at the compensation angle θ_C_. Hence:

$$\theta {*_C}={\theta _C}\forall k.$$

The corresponding maximum end-to-end efficiency is:

η*_total, C_*(k) = η_PA · η_WPT(k)*, evaluated at θ = θ_C_.

Crucially, since the Chireix combiner operates at θ_C_ > 0°, the effective load seen by the combiner differs from that at θ = 0°. This results in a higher *η*_*comb, C*_ at its optimum and, consequently, a superior end-to-end efficiency compared to the Wilkinson combiner under identical coupling conditions.

Table 2 summarizes the optimal operating conditions and corresponding peak efficiencies for representative coupling values.

**Table 2:** Optimal outphasing angle and peak end-to-end efficiency for Wilkinson and Chireix combiners at representative coupling conditions. (η_PA = 0.80, θc = 70°, parameters from Table 1).

k	θ*_W_	η*_total, W_	θ*_C_	η*_total, C_	Gain (C over w)
0.1	0°	≈ 0.38	70°	≈ 0.42	+ 4 pp
0.2	0°	≈ 0.53	70°	≈ 0.61	+ 8 pp
0.3	0°	≈ 0.64	70°	≈ 0.72	+ 8 pp

Note: All values assume η_PA = 0.80 and η_WPT(k) from Eqs. (7)–(8). Figure 3, by contrast, assumes an ideal WPT link (η_WPT = 1.0) to isolate combiner behavior. Both sets are correct for their stated assumptions. ADS simulation (Fig. 7) gives lower absolute values because it reports circuit-level efficiency normalized to source power, excluding the η_PA and η_WPT terms of Eq. (9).

### Circuit-level validation

**Fig. 7:**
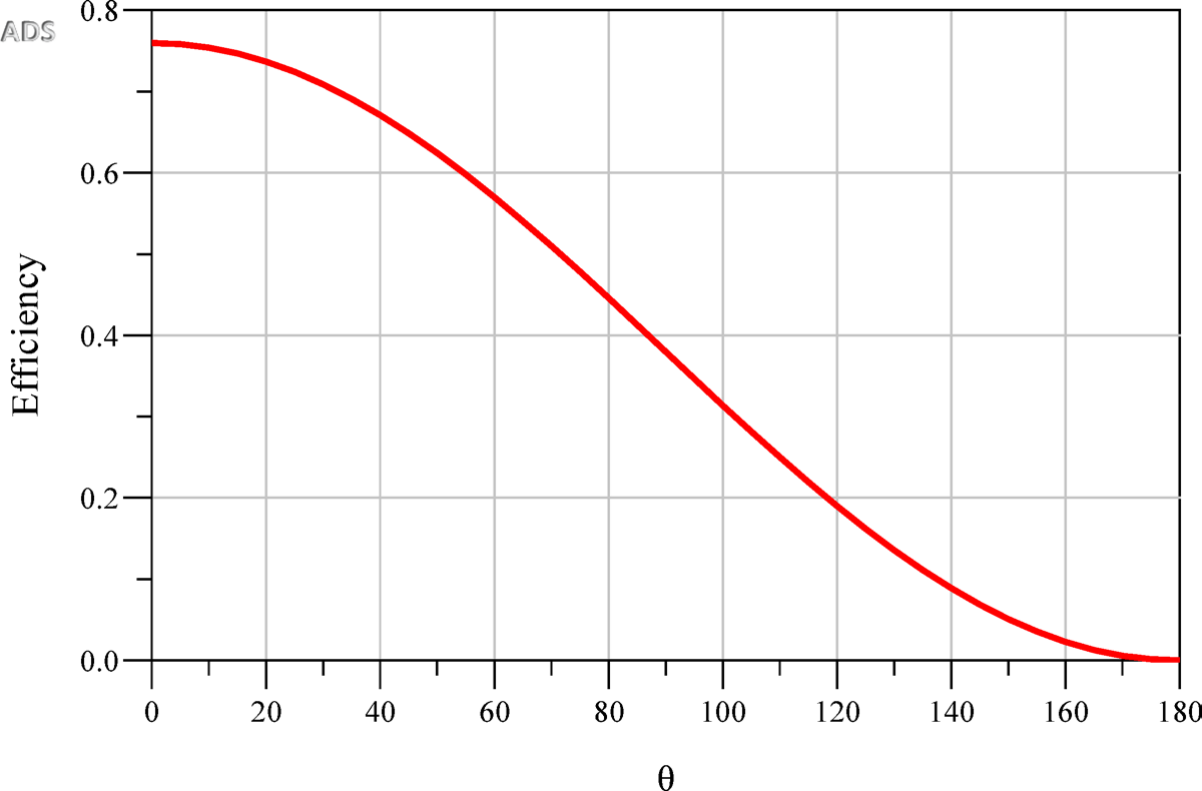
ADS harmonic balance simulation of end-to-end efficiency versus outphasing angle θ for the Wilkinson combiner configuration at 13.56 MHz, showing monotonic efficiency degradation consistent with the analytical model.

To provide circuit-level support for the analytical framework, harmonic balance (HB) simulations were performed in Keysight Advanced Design System (ADS) at 13.56 MHz for the Wilkinson combiner configuration. The simulated system consists of two constant-envelope RF branches combined into a resistive load, consistent with the system-level model described in Section II. The outphasing angle θ was swept from 0° to 180° in steps of 5°, and the end-to-end efficiency was computed as the ratio of power delivered to the load to the total input power from the two RF sources. The ADS simulation confirms the analytically predicted monotonic decrease in efficiency with increasing outphasing angle, consistent with Eq. (5) and the Wilkinson efficiency model (with η_PA = 0.80). At θ = 0°, the simulated efficiency reaches 0.759, corresponding to in-phase combining where maximum power is delivered to the load. At θ = 90°, the efficiency drops to 0.38, reflecting the increasing dissipation associated with out-of-phase power components. The ADS efficiency values in Fig. 7 are lower than the analytical values in Table 2 because the circuit simulation normalizes efficiency to source power and includes practical combining losses, whereas the analytical model applies idealized lossless branch sources. Figure 7 therefore validates the predicted trend rather than the absolute numerical values. Nevertheless, this monotonic degradation trend is in qualitative agreement with the analytical Wilkinson model, which predicts *η*_*comb, W*_ *(θ)* = cos²(θ/2), and confirms that the system-level framework correctly captures the dominant efficiency behavior of isolating combiner architectures under outphasing operation. These results provide independent circuit-level confirmation that the analytical trends presented in Section IV are physically consistent and reproducible in a circuit-level simulation environment, supporting the validity of the proposed framework for early-stage wearable WPT system design.

To further validate the Chireix combiner model,** a corresponding harmonic balance simulation was performed using reactive compensation elements.**

**Fig. 8:**
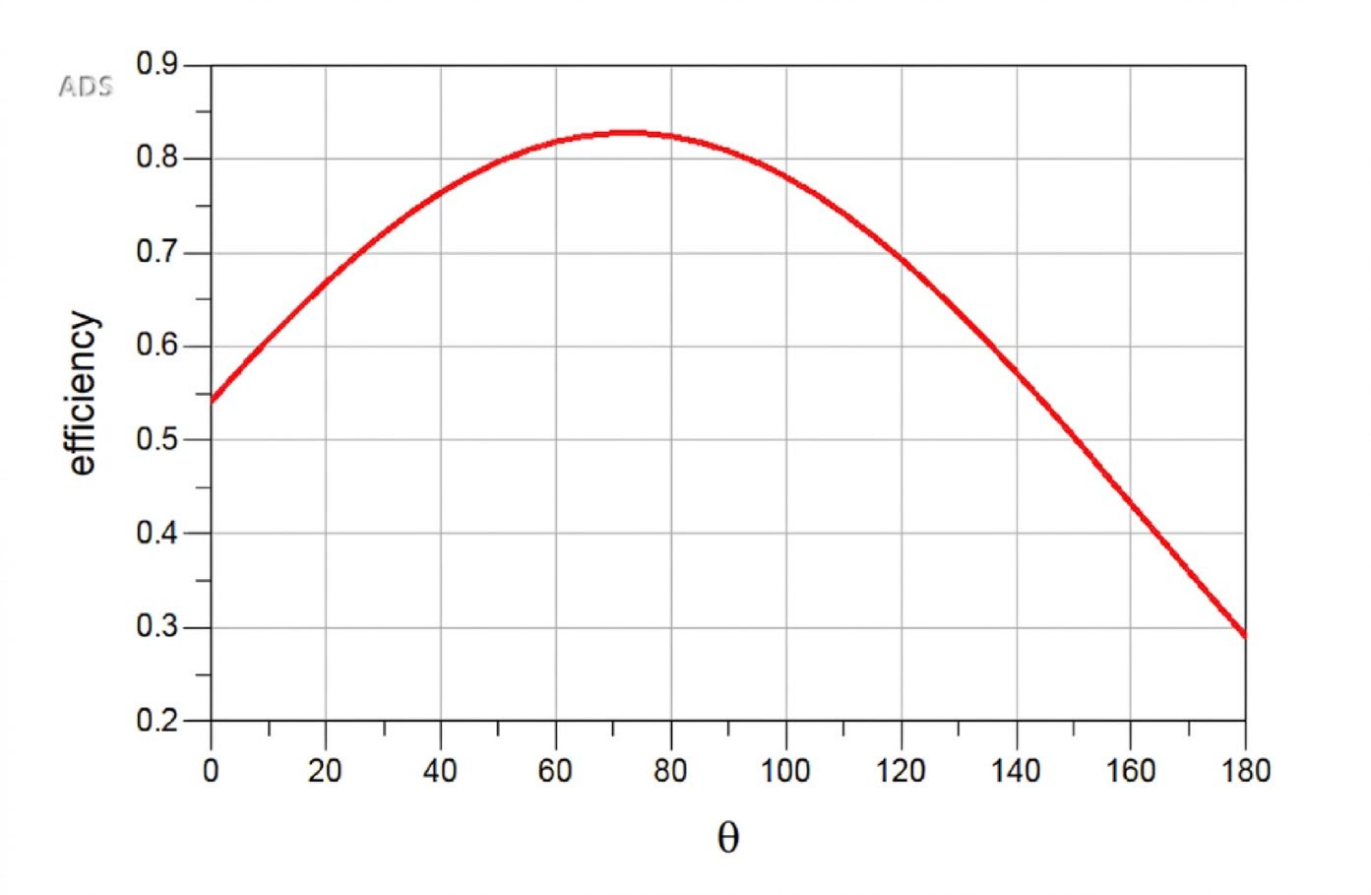
ADS harmonic balance simulation of end-to-end efficiency versus outphasing angle θ for the Chireix combiner configuration at 13.56 MHz. The reactive compensation elements were selected for a nominal compensation angle θ_c = 70°. The simulated efficiency exhibits a clear peak at θ = 72° with a peak efficiency of approximately 82.5%, degrading monotonically on both sides. This close agreement (72° simulated vs. 70° analytical) confirms the predicted Chireix behavior and validates the analytical model presented in Eq. (6).

Figure 8 shows the simulated end-to-end efficiency for the Chireix combiner configuration under the same operating conditions. The reactive compensation elements were selected for a nominal compensation angle θ_c = 70°. The simulated efficiency exhibits a clear peak at θ = 72° with a peak efficiency of approximately 82.5%, degrading monotonically on both sides of the peak. This behavior qualitatively matches the analytical Chireix model presented in Eq. (6) and confirms that reactive load modulation can be realized in a practical circuit implementation. The slight difference between the analytical peak (70°) and simulated peak (72°) is within expected tolerance for practical circuit implementations and does not affect the qualitative validation of the Chireix efficiency model. Together with the Wilkinson validation in Fig. 7, these circuit-level results substantiate the analytical framework and support its use for system-level design exploration.

## Discussion

### Implications for wearable wpt system design

The results presented in Section IV highlight several important implications for the design of wearable wireless power transfer (WPT) systems employing outphasing power amplifier (PA) transmitters. Most notably, the analysis demonstrates that transmitter-side efficiency optimization alone is insufficient to guarantee high end-to-end efficiency in realistic wearable scenarios. Instead, system performance is strongly governed by the interaction between PA back-off operation, power combiner behavior, and coupling-dependent wireless link conditions.

In wearable applications, variations in coil alignment, separation distance, and orientation are unavoidable due to user motion. As shown in Sections IV.C and IV.D, these variations directly affect the coupling coefficient and, consequently, the effective load impedance seen by the transmitter. Under weak or fluctuating coupling conditions, improvements achieved through advanced power combining techniques are constrained by the wireless link itself. This observation emphasizes that robust coupling management and coil placement strategies are at least as critical as PA efficiency enhancement when targeting reliable wearable WPT operation.

The choice of power combiner topology also carries important design implications. Wilkinson combiners offer predictable and stable performance across a wide range of operating conditions, making them suitable for wearable systems where coupling conditions cannot be tightly controlled. Their isolating nature reduces sensitivity to load variations, at the expense of reduced efficiency under deep back-off. In contrast, Chireix combiners can provide superior efficiency near a targeted back-off region when coupling conditions are sufficiently strong and stable. However, their sensitivity to load reactance requires careful coordination between outphasing control and expected coupling behavior.

From a system-design perspective, these results suggest that wearable WPT transmitters should be designed with an awareness of the expected coupling envelope, rather than optimized solely for peak efficiency under ideal conditions. In scenarios where coupling is highly variable, prioritizing robustness through isolating combiners and conservative outphasing ranges may yield more consistent performance. Conversely, applications with controlled coil positioning or constrained motion may benefit from reactive compensation techniques that improve efficiency at moderate back-off.

Overall, the findings reinforce the importance of holistic co-design in wearable WPT systems. Effective solutions require simultaneous consideration of PA architecture, power combiner topology, outphasing control strategy, and wireless link characteristics. By providing a system-level framework that captures these interactions, the proposed analysis offers practical guidance for selecting transmitter architectures that balance efficiency, robustness, and adaptability in real-world wearable applications. Based on the presented analysis, the following practical design guidelines are recommended: (1) For highly variable coupling conditions (k < 0.15), the Wilkinson combiner is preferred due to its robustness and predictable efficiency; (2) For moderate-to-strong coupling (k ≥ 0.2) with frequent moderate back-off operation, the Chireix combiner with θc tuned to 60°–70° offers superior average efficiency; (3) Outphasing angle should be restricted to 0°–90° in dynamic wearable scenarios to avoid deep back-off regions where combiner losses dominate.

### Trade-off between efficiency and robustness

The comparative analysis of Wilkinson and Chireix power combiners reveals a fundamental trade-off between efficiency optimization and operational robustness, which is particularly relevant in wearable wireless power transfer (WPT) systems. While both architectures enable outphasing-based back-off operation, their efficiency characteristics and sensitivity to load variations differ substantially, leading to distinct design implications.

The Wilkinson combiner prioritizes robustness through its isolating structure. By dissipating out-of-phase power in the isolation resistor, it decouples the branch amplifiers from load impedance variations introduced by the wireless link. As a result, system behavior remains predictable across a wide range of outphasing angles and coupling conditions. Although this approach limits peak efficiency at moderate and deep back-off, it provides stable performance in scenarios where coupling variations are frequent or difficult to control, such as during user motion or misalignment in wearable applications.

In contrast, the Chireix combiner exploits reactive load compensation to enhance efficiency near a specific back-off operating point. When the outphasing angle is close to the compensation angle and coupling conditions are favorable, reactive power circulation is minimized and higher efficiency can be achieved. However, this efficiency gain comes at the cost of increased sensitivity to coupling-dependent load variations. Deviations from the compensated condition, either due to changes in outphasing angle or wireless link characteristics, lead to a more pronounced efficiency degradation than observed in isolating architectures.

This trade-off highlights an important system-level consideration: maximizing peak efficiency does not necessarily yield optimal average performance in dynamic environments. In wearable WPT systems, where operating conditions fluctuate continuously, architectures that offer moderate but stable efficiency may outperform designs that achieve higher efficiency only under narrowly defined conditions. Consequently, the selection of combiner topology should be guided by the expected variability of the wireless link rather than by peak-efficiency metrics alone.

The presented results suggest that hybrid design strategies may be beneficial in practice. For example, restricting the operating range of the outphasing angle, adaptively selecting compensation points, or combining robust combiner architectures with coupling-aware control strategies could help balance efficiency and robustness. While such approaches are beyond the scope of the present study, the system-level framework developed here provides a suitable foundation for evaluating these trade-offs during early-stage design.

Overall, the efficiency–robustness trade-off identified in this work reinforces the need for context-aware transmitter design in wearable WPT applications. By explicitly quantifying how power combiner behavior interacts with coupling variability, the analysis enables informed architectural choices that align performance objectives with realistic operating conditions.

### Model scope and limitations

The proposed analysis is intentionally developed as a system-level modeling framework aimed at capturing dominant efficiency trends and design trade-offs in outphasing wireless power transfer (WPT) transmitters. As such, several modeling assumptions are adopted to maintain analytical transparency and computational efficiency. While these assumptions are appropriate for the objectives of this work, they also define the scope and limitations of the presented results.

First, the power amplifier (PA) branches are modeled as ideal constant-envelope voltage sources operating with fixed intrinsic efficiency. This abstraction neglects device-level nonlinearities, finite switching losses, and efficiency variations caused by load impedance changes. In practical implementations, such effects may influence absolute efficiency values, particularly under deep back-off or extreme load conditions. However, the adopted assumption is consistent with the goal of isolating relative efficiency trends associated with power combining and wireless link behavior, which are shown to dominate system-level performance in wearable scenarios.

Second, losses associated with matching networks, passive components, and interconnects are not explicitly modeled. Including these effects would reduce absolute efficiency but would not alter the qualitative trade-offs identified between isolating and non-isolating combiner architectures. As a result, the conclusions drawn in this work remain valid for comparative analysis and early-stage architecture selection, where relative performance trends are of primary interest.

Third, the wireless power transfer link is represented using a lumped-element inductive model that captures coupling-dependent reflected impedance but does not account for detailed electromagnetic field distributions or coil geometry optimization. This choice is well suited for analyzing coupling-induced load modulation and its interaction with transmitter behavior, but it does not address aspects such as spatial field shaping, coil parasitics, or compliance with electromagnetic exposure constraints. These considerations are more appropriately addressed through full-wave simulations or experimental characterization at later stages of the design process.

Finally, the Chireix combiner efficiency is modeled using a phenomenological expression that captures the efficiency envelope around the compensation angle rather than detailed circuit-level behavior. This abstraction enables efficient exploration of back-off efficiency trends and sensitivity to coupling variations, but it does not represent frequency-dependent effects or harmonic interactions that may arise in practical implementations.

Despite these limitations, the presented framework provides meaningful and actionable insight into the interaction between outphasing control, power combiner topology, and coupling-dependent wireless loads. The model is particularly valuable during early design stages, where rapid evaluation of architectural choices is required before committing to detailed circuit design or experimental prototyping. Future work may extend the framework by incorporating measured PA efficiency characteristics, frequency-dependent combiner models, or experimentally validated WPT links to further refine quantitative predictions while preserving the system-level perspective established in this study. Additionally, the model assumes ideal voltage sources and does not account for potential detuning effects in the WPT link due to coupling variations, which could be explored in future refinements.

### Positioning with respect to prior work

The contribution of this work can be clearly understood when positioned relative to existing research on outphasing power amplifiers and wireless power transfer (WPT) systems. Prior studies on outphasing and load-modulated power amplifiers have primarily focused on improving back-off efficiency through circuit-level innovations, reactive compensation techniques, or broadband combiner design, typically assuming fixed and resistive load conditions^10,12,14,25–27^. While these works provide valuable insight into transmitter efficiency optimization, they do not account for the dynamic and reactive loading conditions introduced by inductive wireless links, particularly in wearable applications.

Conversely, a substantial body of literature on WPT systems has concentrated on coil design, electromagnetic optimization, and link-level efficiency analysis under varying alignment and coupling conditions^4–6,18,21,23,24^. In many of these studies, the transmitter is modeled as an ideal RF source, and the interaction between transmitter architecture, power combining behavior, and coupling-dependent load modulation is not explicitly considered. As a result, the implications of transmitter-side back-off operation and combiner selection on end-to-end WPT efficiency are often overlooked.

More recent system-level investigations have begun to emphasize co-design approaches for RF transmitters and WPT links, highlighting the importance of considering interactions between circuit behavior and wireless coupling conditions^22,23,32^. However, these studies typically address conventional transmitter architectures or do not explicitly examine outphasing operation and power combiner trade-offs under dynamic coupling scenarios.

The present work complements and extends prior research by providing a unified system-level framework that explicitly couples outphasing PA operation, power combiner behavior, and coupling-dependent WPT load variations. By integrating analytical models for Wilkinson and Chireix combiners with a lumped-element WPT representation, the proposed approach bridges the gap between PA-centric efficiency analysis and wireless link modeling. This joint perspective enables identification of efficiency trends and design trade-offs that are not apparent when transmitter and wireless subsystems are analyzed in isolation.

Importantly, the focus of this work is not on achieving record efficiency through circuit-level optimization, but rather on understanding how architectural choices influence end-to-end performance under realistic wearable conditions. In this context, the presented framework provides complementary insight to detailed circuit and electromagnetic studies, offering a valuable tool for early-stage design and architecture selection. By emphasizing relative efficiency trends, robustness considerations, and coupling-aware behavior, the analysis aligns well with the growing need for system-level co-design methodologies in wearable and low-power wireless applications.

Overall, this work advances the state of the art by demonstrating that outphasing transmitter optimization must be evaluated within the broader context of wireless power delivery, and that meaningful efficiency gains can only be realized through coordinated consideration of PA control, power combiner topology, and wireless coupling dynamics.

## Conclusion

This work presented a system-level analytical framework for outphasing power amplifier transmitters driving inductive wireless power transfer (WPT) links under realistic wearable operating conditions. By jointly modeling outphasing PA operation, Wilkinson and Chireix combiner behavior, and coupling-dependent reflected impedance, the framework enables closed-form derivation of optimal operating conditions — a capability not available when transmitter and wireless subsystems are analyzed in isolation.

The key analytical result is the systematic characterization of the optimal outphasing angle θ*(k) as a function of coupling: for the Wilkinson combiner, θ* = 0° for all coupling conditions, with achievable efficiency determined entirely by the wireless link; for the Chireix combiner, peak efficiency occurs at the compensation angle θ_C_ = 60°–70° regardless of coupling, but its magnitude is strongly modulated by k. At moderate coupling (k = 0.2), the Chireix architecture outperforms Wilkinson by up to 8% points at the optimal operating point. Under weak or rapidly varying coupling (k < 0.15), the Wilkinson combiner provides superior robustness and more consistent average efficiency.

These analytical trends were further corroborated by ADS harmonic balance circuit simulations at 13.56 MHz for both combiner topologies, confirming the predicted monotonic Wilkinson efficiency degradation and the characteristic Chireix efficiency peak near θ_c = 70° at circuit level.

These findings establish three concrete design guidelines for wearable WPT systems: (1) use Wilkinson combiners when coupling variability is high (k < 0.15); (2) use Chireix combiners with θ_C_ = 60°–70° when moderate-to-strong coupling is available (k ≥ 0.2); and (3) restrict the outphasing range to 0°–90° in dynamic scenarios to avoid deep back-off where combiner losses dominate.

Future work will extend this framework by incorporating measured PA efficiency characteristics, frequency-dependent combiner models, and experimental validation under dynamic wearable scenarios, further bridging system-level modeling and practical hardware implementation.

## Data Availability

All data generated or analysed during this study are included in this published article.
